# Pharmacological Blockade of NLRP3 Inflammasome/IL-1β-Positive Loop Mitigates Endothelial Cell Senescence and Dysfunction

**DOI:** 10.14336/AD.2021.0617

**Published:** 2022-02-01

**Authors:** Alejandra Romero, Pilar Dongil, Inés Valencia, Susana Vallejo, Álvaro San Hipólito-Luengo, Guillermo Díaz-Araya, José L Bartha, María M González-Arlanzón, Fernando Rivilla, Fernando de la Cuesta, Carlos F Sánchez-Ferrer, Concepción Peiró

**Affiliations:** ^1^Department of Pharmacology and Therapeutics, School of Medicine, Universidad Autónoma de Madrid, Madrid, Spain.; ^2^Instituto de Investigación Sanitaria del Hospital Universitario La Paz (IdiPAZ), Madrid, Spain.; ^3^PhD Programme in Pharmacology and Physiology, Doctoral School, Universidad Autónoma de Madrid, Madrid, Spain.; ^4^Department of Pharmacological and Toxicological Chemistry, Faculty of Chemical and Pharmaceutical Sciences, Universidad de Chile, Santiago, Chile.; ^5^Advanced Center for Chronic Diseases (ACCDiS), Faculty of Chemical and Pharmaceutical Sciences and Faculty of Medicine, Universidad de Chile, Santiago, Chile.; ^6^Department of Obstetrics and Gynecology, School of Medicine, Universidad Autónoma de Madrid, Madrid, Spain.; ^7^Division of Pediatric Surgery, Hospital Universitario Ramón y Cajal, Madrid, Spain.

**Keywords:** Interleukin-1β, NLRP3 inflammasome, endothelial cell, senescence, vascular dysfunction, angiotensin-(1-7), klotho

## Abstract

The clinical relevance of IL-1β in chronic inflammation underlying atherosclerosis has been reinforced by recent evidence associating pharmacological inhibition of the cytokine with lower cardiovascular risk. Previously, we have demonstrated a direct involvement of IL-1β in endothelial senescence. Therefore, this can be a key mechanism contributing to the sterile inflammatory milieu associated with aging, termed inflammaging. In the present study, we have evaluated whether a positive feedback of IL-1β in the NLRP3 inflammasome via NF-κB could promote human endothelial senescence *in vitro* and murine endothelial dysfunction *in vivo*. Our results indicate that the NLRP3 inflammasome is pivotal in mediating the detrimental effects of IL-1β, showing that auto-activation is a crucial feature boosting endothelial cell senescence *in vitro*, which is paralleled by vascular dysfunction *in vivo*. Hence, the inhibitor of NLRP3 inflammasome assembly, MCC 950, was able to disrupt the aforementioned positive loop, thus alleviating inflammation, cell senescence and vascular dysfunction. Besides, we explored alternative NLRP3 inflammasome inhibitory agents such as the RAS heptapeptide Ang-(1-7) and the anti-aging protein klotho, both of which demonstrated protective effects *in vitro* and *in vivo*. Altogether, our results highlight a fundamental role for the hereby described NLRP3 inflammasome/IL-1β positive feedback loop in stress-induced inflammaging and the associated vascular dysfunction, additionally providing evidence of a potential therapeutic use of MCC 950, Ang-(1-7) and recombinant klotho to block this loop and its deleterious effects.

Vascular aging is a multifaceted and complex process that ultimately renders the vessels prone to profound functional and structural disturbances that favor cardiovascular disease. In fact, vascular aging is a main biomarker of frailty and a common feature of most age-associated diseases [1, 2].

One of the main mechanisms contributing to vascular aging is endothelial cell senescence. Senescent cells undergo functional and morphological changes that ultimately lead to growth arrest while remaining metabolically active. Importantly, these cells acquire a senescence-associated secretory phenotype (SASP), which results in the over-production and release of a wide array of cytokines and chemokines [3, 4]. The SASP favors leukocyte recruitment and is considered a main driver of sterile age-related inflammation or “inflammaging” [5]. Functionally, endothelial cell senescence is tightly associated with endothelial dysfunction and defective vasodilatation, considered as early markers of vascular disease and atherosclerosis [3, 6]. Thus, understanding the basis of endothelial cell senescence is crucial to identify therapeutic approaches that may attenuate this phenomenon and its deleterious consequences.

Premature endothelial cell senescence can be triggered by a variety of extracellular stressors. Among them, the pro-inflammatory cytokine interleukin (IL)-1β has recently been identified as a key player in human vascular disease, upon the demonstration by the CANTOS trial that pharmacological IL-1β blockade reduces the incidence of atherothrombotic events [7]. These results have been considered as a proof-of-concept of the inflammatory development of atherosclerosis.

IL-1β is the main product of the activation of the multi-complex protein nucleotide-binding oligo-merization domain leucine-rich repeat and pyrin domain containing-3 (NLRP3) inflammasome, which activates caspase-1 to transform pro-IL-1β into mature IL-1β. The NLRP3 inflammasome is part of the physiological innate immune response, but its overactivation causes a potent self-amplifying response, which might cause damage and contribute to several pathologies, including vascular diseases, if not properly controlled [8, 9]. Our group has recently proved that IL-1β induces human endothelial cell senescence [10]. However, whether IL-1β itself can activate NLRP3 inflammasome and whether such activation may be involved in endothelial cell senescence and dysfunction is yet to be established.

In the search of anti-senescence agents, we have recently reported the protective capacity of the heptapeptide angiotensin (Ang)-(1-7) [10]. Ang-(1-7) belongs to the protective branch of the renin-angiotensin system (RAS), where it counteracts Ang II, the main bioactive peptide of the RAS and a key player in human vascular disease [11]. Ang-(1-7) was shown to exert anti-senescence actions on human endothelial cells via the upregulation of the anti-aging protein klotho [10]. However, the intracellular mechanisms by which Ang-(1-7) may exert its protective action are only partially understood.

In this study, we evaluated if there is positive feedback of IL-1β on the NLRP3 inflammasome and whether this loop could promote human endothelial senescence *in vitro*, and endothelial dysfunction *in vivo*, using a murine experimental model. Moreover, we tested the capacity of Ang-(1-7) and klotho to interfere with the IL-1β-NLRP3 inflammasome loop and its deleterious vascular consequences.

## MATERIALS AND METHODS

### Materials

M199 culture medium, foetal calf serum (FCS) was from Biological Industries (Beit-Hamek, Israel). Heparin, endothelial cell growth supplement (ECGS), amphotericin, type II collagenase, type I collagen, EDTA, sodium orthovanadate, phenylmethylsulfonyl fluoride (PMSF) were purchased from Sigma (St. Louis, MO, USA). IL-1β and human recombinant klotho (r-klotho) were purchased from Preprotech (London, UK) and Abcam (ab84072; Cambridge, UK), respectively, while Ang-(1-7) was purchased from Bachem (Bubendorf, Switzerland). The sodium salt CP-456773 (also known as MCC 950) was purchased from Sigma (St. Louis, MO, USA). Anakinra was obtained from Biovitrum (SOBI, Stockholm, Sweden).

### HUVEC isolation and cell culture

Human umbilical vein endothelial cells (HUVEC) were isolated from umbilical cords from donors at Hospital Universitario La Paz (Spain, Madrid) with informed consent, as previously described [10]. All procedures followed the Spanish legislation and were under approval of the appropriate Ethics Committee. HUVEC were isolated by chemical digestion with type II collagenase (2 mg/ml), and cultured in M199 medium supplemented with 20 % FCS, 25 μg/ml ECGS, 100 μg/ml heparin and antibiotics (100 U/ml penicillin, 100 µg/ml streptomycin and 2.5 µg/ml amphotericin B) at 37°C in a humidified atmosphere with 5 % CO_2_. For all experiments, cells at passages 1-5 were treated for 18-24 h as appropriate.

### Senescence Associated-β-Galactosidase assay

Senescence Associated-β-Galactosidase (SA-β-gal) staining was performed using a commercial kit from Sigma (St. Louis, MO, USA), as previously described [10]. The percentage of SA-β-gal positive cells stained in blue over total cells was determined by blind manual scoring of at least 1,000 cells per sample in 12 randomized fields per condition, under an inverted microscope Nikon Eclipse T300 (Tokyo, Japan) in phase contrast mode with a 20X objective.

### Western blotting

HUVEC were treated as indicated, lysed and their protein content was quantified by the bicinchoninic acid (BCA) method (Thermo Fisher Scientific, Illinois, USA). Thereafter, 20 μg of protein lysates were separated by SDS-PAGE electrophoresis and later transferred to polyvinyl membranes (Merck, Darmstadt, Germany). Proteins were detected as previously described [10]. Primary antibodies against NLRP3 (20B-0012; Adipogene, Switzerland; 1:1,000), IL-1β/IL-1F2 (AF-401-NA; R&D System, USA; 1:1,000), p21 (sc-6246; Santa Cruz Biotechnology, USA; 1:1,000), phospho-p65 (p-p65; (S536; Cell Signaling, USA; 1:1,000) and p65 (B14E12; Cell Signaling, USA; 1:1,000) were used, followed by incubation with corresponding horseradish peroxidase-conjugated secondary antibodies (Bio-Rad; 1:10,000). Protein levels were normalized to β-Actin signal (Sigma-Aldrich; 1:10,000). Immunoreactive bands were detected using an enhanced chemiluminescence ECL detection kit (Bio-Rad, California, USA) and quantified by densitometry using ImageJ 1.51w free software.

### Determination of NLRP3 inflammasome activation by indirect immunofluorescence

Upon NLRP3 inflammasome activation, one of its components, the ASC protein assembles and forms toroidal structures known as specks [12]. The ASC specks were visualized in HUVEC by indirect immunofluorescence, as previously described [13], after incubation with a primary anti-ASC antibody (ADI-905-173; Enzo Life Science, Switzerland; 1/250), followed by an Alexa Fluor 647-conjugated goat anti-rabbit IgG secondary antibody (Jackson Immuno Research, Cambridge, UK). Nuclei were counterstained with 1 μmol/l 4’-6’-diamidino-2-phenylindole (DAPI) (Molecular Probes-Invitrogen Corporation; USA). ASC specks per field were quantified by manual blind scoring of 18 radial distributed fields per sample under an inverted microscope Eclipse TE300 (Nikon). Representative images were acquired with a TCS SPE confocal microscope (Leica, Wetzlar, Germany).

### Determination of IL-1β secretion by ELISA

After the appropriate treatments and incubation times, supernatants were collected, centrifuged at 900 g for 10 min at 4°C and frozen at -20ºC until further use. IL-1β was measured with an ELISA immunoassay (Raybiotech, Norcross, GA, USA) according to the manufacturers' instructions.

### In vivo experimental treatments

Four-month-old male C57BL6/J mice were maintained under standardized conditions with an artificial 12 h-12 h dark-light cycle, and ad libitum access to food and water. All animal studies were performed in accordance with National and European guidelines and regulations (RD 53/2013; Directive 2010/63/EU) and were approved by the institutional animal care (CEI-59-1052-A062; PROEX 026/15).

Mice were randomly allocated to the different experimental groups. Osmotic mini-pumps (Alzet, model 1007D, DURECT Corporation, Cupertino, CA, USA) were implanted subcutaneously under the scapule in animals previously anesthetized with i.p. injection of 50 μL Imalgene© (ketamine, 50 mg/mL) and 10 μL de xilacine (Xilagesic 2%) solved in 0.5 mL NaCl (0.9%). The mini-pumps contained a volume of 100 μL and a constant infusion speed of 0.5 μL/hour was established. Mice were infused for 7 days either with saline vehicle (NaCl 0.9%) or interleukin-(IL)-1β (12 μg/kg/day). Some animals were also treated with one of the following drugs: anakinra (i.p. injection of 100 mg/kg on days 4, 5, and 6); MCC 950 (i.p. injection of 10 mg/kg on days 2, 4, and 6); Ang-(1-7) (0.58 mg/kg/day by minipump infusion) or r-klotho (i.p. injection of 0.02 mg/kg on days 2, 4, and 6). The doses and administration regimes of the different drugs were chosen based on previous studies by us and others [9, 13, 14]. On day 0 and 7, weight, plasma glucose and arterial pressure were determined. At the end of the treatment period, animals were sacrificed by exposition to carbon dioxide and mesenteric arteries and aortas were extracted for vascular reactivity and gene expression analysis, respectively, as described below.

### Microvascular reactivity

For reactivity experiments, segments from first branch mesenteric arteries (internal diameter 150-200 µm) were mounted on a small vessel myograph (DMT, Denmark) to measure isometric tension, as described before [14]. Arteries were maintained in Krebs-Henseleit solution at physiologic conditions (37ºC, continuous bubbling with 95 % O_2_ - 5 % CO_2_ mixture and pH 7.4). Arteries were contracted with 3 µM noradrenaline (NA) and then the vasoactive responses to cumulative concentrations of acetylcholine (ACh; 0.1 nM to 10 µM) were tested. In some experiments, the mesenteric segments were preincubated for 30 min with MCC 950 (10 µmol/l) or anakinra (100 μg/ml) before the addition of NA.

### Total RNA isolation and quantitative real-time (qRT)-PCR

For gene expression analysis, total RNA was extracted from mice aortas with a commercial kit “RNAeasy Mini Kit” (Qiagen, Hilden; Germany) according to manufacturer´s instructions. RNA integrity was tested by a NanoDrop 2000 spectrophotometer (Thermo Fisher Scientific) and cDNA synthesis was performed using the First-Strand cDNA Synthesis kit (NZYTech), with 500 ng of RNA as template and following the manufacturer’s instructions.

**Figure 1.**
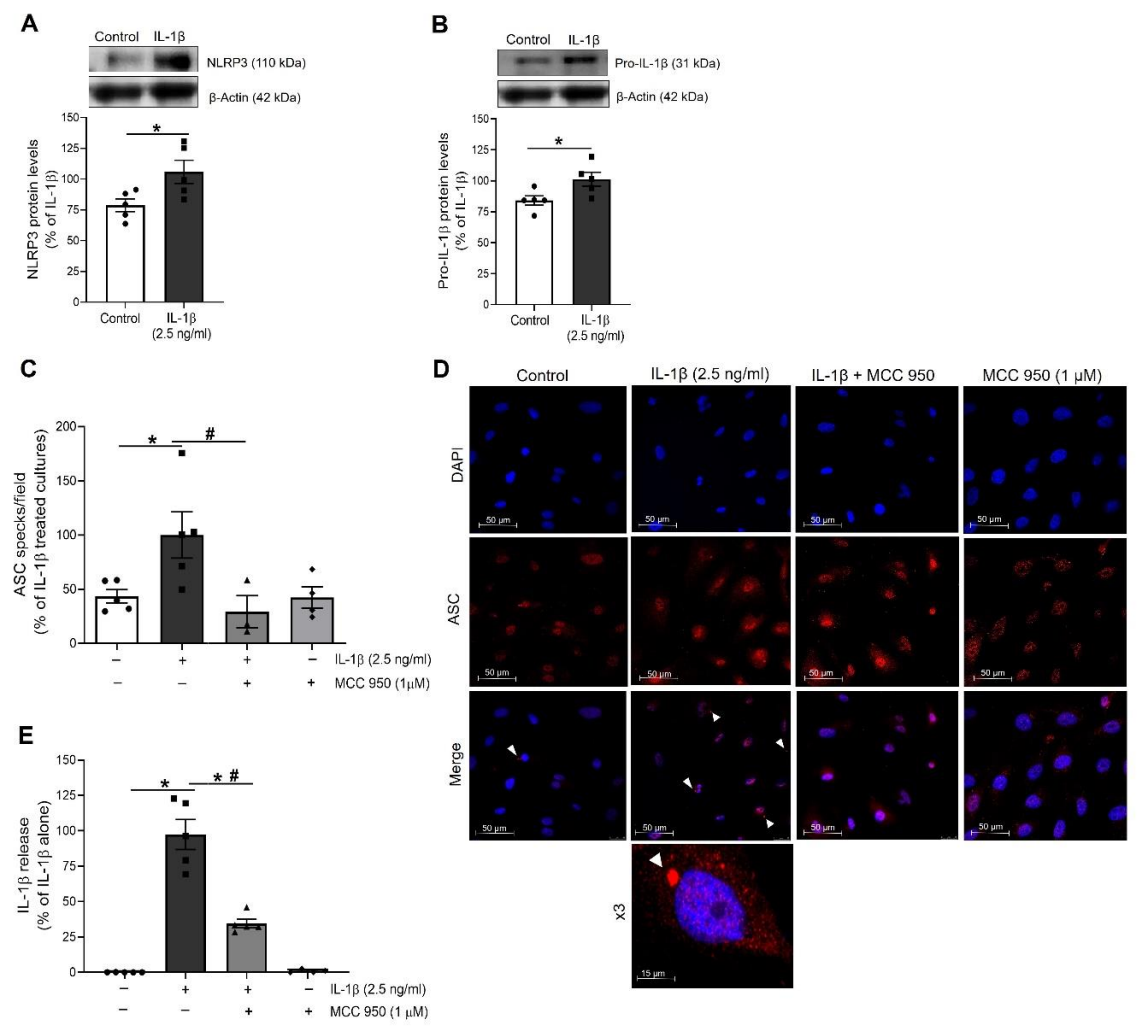
IL-1β promotes priming and activation of the NLRP3 inflammasome in HUVEC. The expression of the NLRP3 inflammasome components (A) NLRP3 and (B) pro-IL-1β was determined by Western blot in total lysates from HUVEC stimulated for 18-24 h with IL-1β. Representative blots are shown on top of the corresponding graphs, where β-Actin was employed as loading control (n= 4-5; *p<0.05 vs. untreated cells by unpaired t-test). (C) The NLRP3 inflammasome activation was determined by the observation of ASC specks by indirect immunofluorescence after treatment with IL-1β alone or in presence of MCC 950 (1 µM). The percentage of ASC specks per cell culture field was quantified under a fluorescence microscope (n= 3-4; *p<0.05 vs. untreated cells; #p<0.05 vs. IL-1β-treated cells, by two-way ANOVA and Tukey post-hoc test). (D) Confocal representative photographs (630X magnification) where white arrowheads show the location of toroidal-shaped ASC specks as detected by a specific antibody against ASC (red), while cell nuclei were counterstained with DAPI (blue). (E) IL-1β, the final product of the NLRP3 inflammasome activation was measured in the supernatant of treated cells by ELISA (n= 4-5; *p<0.05 vs. untreated cells; #p<0.05 vs. IL-1β-treated cultures levels by two-way ANOVA and Tukey post-hoc test). HUVEC were treated with 2.5 ng/ml IL-1β in all experiments. All data are shown as mean ± SEM and expressed as percentage of IL-1β-induced levels.

qRT-PCR reactions were performed with iTaq Universal SYBR Green Supermix (Bio-Rad) on a 7500 Fast Real-Time PCR System (Thermo Fisher Scientific) and specific primers (Sigma) against NLRP3, klotho and p53 (see primers in Supplementary methods Table 1). The PCR conditions were 95 ºC for 20 s, followed by 40 cycles at 95 ºC for 3 s and 60 ºC for 30 s. The relative quantification of gene expression was determined by 2^-ΔΔCt^ method and *18S* housekeeping gene was used for normalizing.

### Statistical analysis

Data are expressed as mean ± standard error of the mean (SEM) for the indicated numbers of independent experiments. Statistical analysis was performed using GraphPad, Prism 8.0.2 software (California, USA). Firstly, normality was checked for each variable by Shapiro-Wilk test. Two-tailed unpaired Student’s t-test to determine differences between two groups and two-way analysis of variance (ANOVA) followed by Fisher’s LSD test or Tukey post-hoc test when there were more than two groups, were applied as appropriate. For correlations, the Pearson coefficient was determined. A *p* value ≤ 0.05 was considered statistically significant.

**Figure 2.**
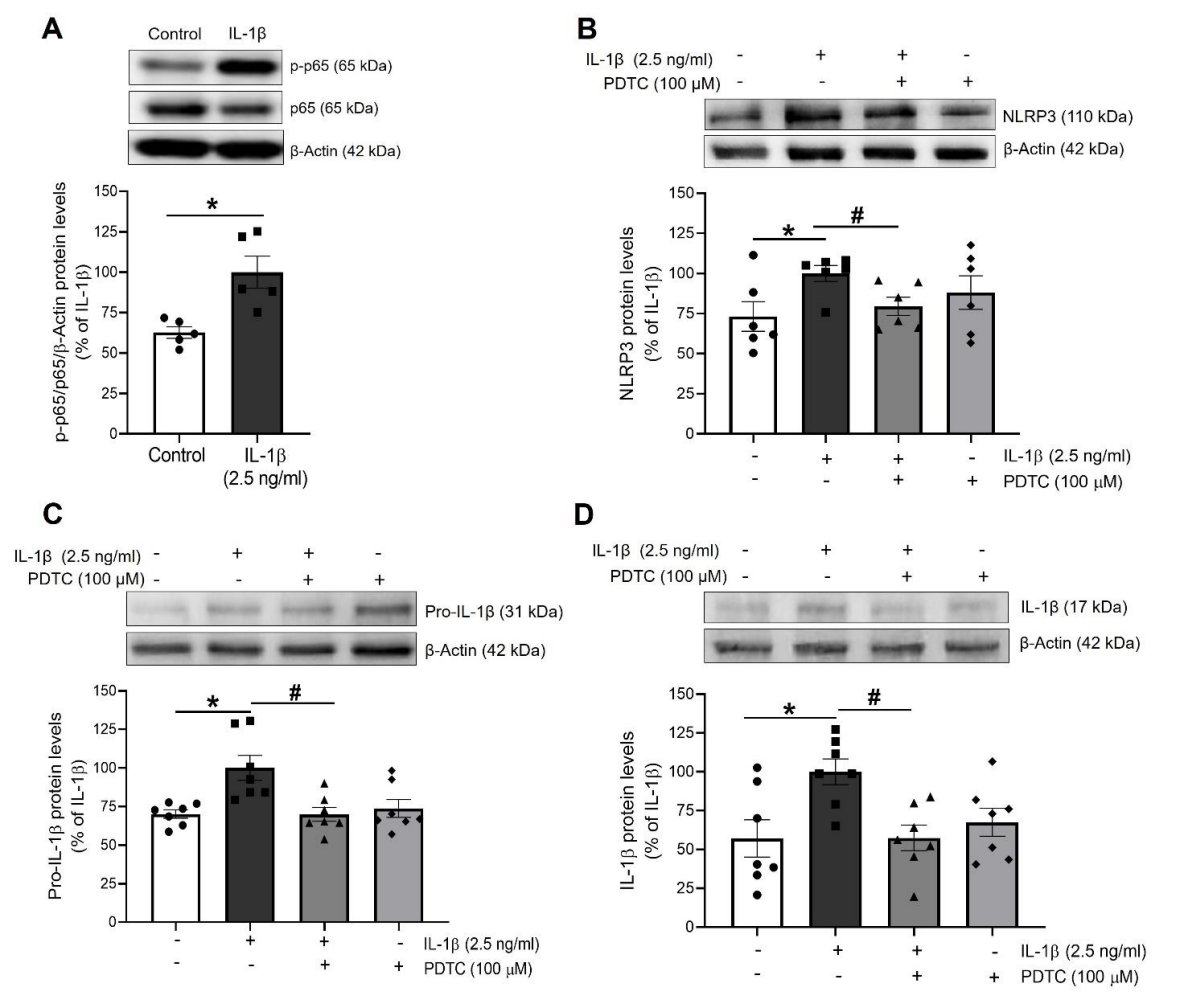
The activation of NLRP3 by IL-1β is mediated by NF-κB. The expression of (A) phosphorylated p65 (p-p65) and total p65 (p65), used as a marker of NF-κB activation, and the NLRP3 inflammasome components (B) NLRP3, (C) pro-IL-1β and (D) mature IL-1β levels was determined by Western blot in total lysates from HUVEC stimulated for 18 h with IL-1β in presence or absence of PDTC (100 µM). Representative blots are shown on top of the corresponding graphs, where β-Actin was employed as loading control (n= 5-7; *p<0.05 vs. untreated cells; #p<0.05 vs. IL-1β-treated cells, by two-way ANOVA and Tukey post-hoc test). All data are shown as mean ± SEM and expressed as percentage of IL-1β-induced levels.

**Figure 3.**
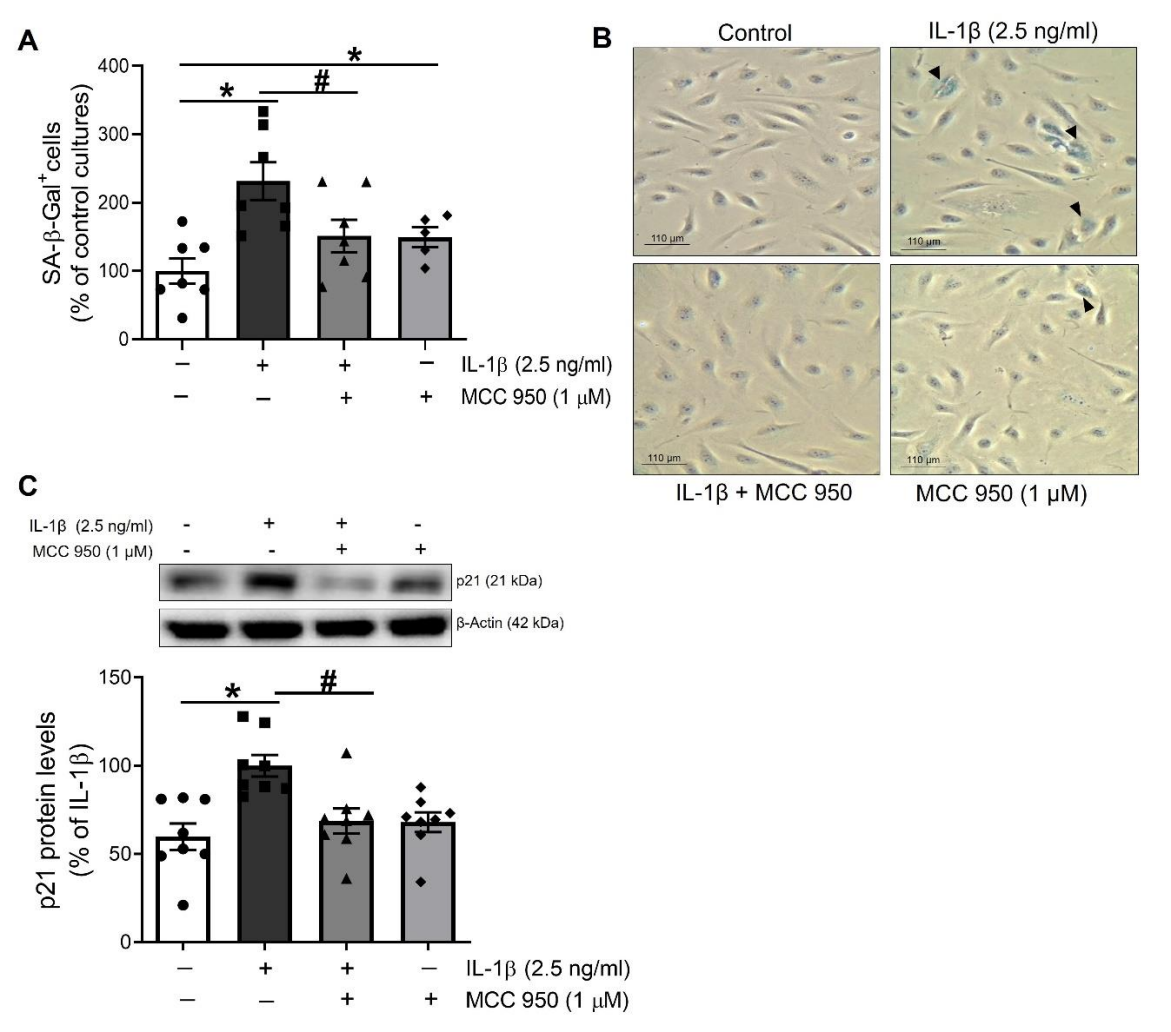
The inflammasome NLRP3 is implicated in the pro-senescence effect of IL-1β in endothelial human cells. HUVEC were treated with IL-1β (2.5 ng/ml) alone or in presence of the NLRP3 inflammasome inhibitor MCC 950 (1 μM) for 18-24 h. (A) SA-β-Gal positive stained cells were quantified by manual scoring by a blind observer after the indicated treatments. (B) Representative phase-contrast images are shown SA-β-Gal positive cells stained in blue (black arrowheads). Data are expressed as percentage of the number of senescent cells stained in non-treated cells (n= 5-7; *p<0.05 vs. untreated cells; #p<0.05 vs IL-1β-treated cultures levels by two-way ANOVA and Tukey post-hoc test). (C) The expression of p21 was determined by Western blot in total lysates from HUVEC. A representative blot is shown on top of each graph, where β-Actin was employed as loading control (n= 6-8; *p<0.05 vs. untreated cells; #p<0.05 vs. IL-1β-treated cells, by two-way ANOVA and Tukey post-hoc test). All data are shown as mean ± SEM.

## RESULTS

### IL-1β induces the expression and activation of the inflammasome NLRP3 multi-complex protein in HUVEC

Firstly, we assessed whether IL-1β, the ultimate product of the NLRP3 inflammasome, was able to promote a feedback loop by up-regulating the expression and activation of this multi-complex protein in HUVEC. The NLRP3 inflammasome requires of two signals for its activation [15]. The first signal is known as priming and it is characterized by the expression of some necessary components for the assembly of NLRP3 inflammasome. In HUVEC treated for 18 h with IL-1β (2.5 ng/ml), we observed a stimulation of the priming phase, as evidenced by an increased expression of the NLRP3 (Fig. 1A) and pro-IL-1β (Fig. 1B) inflammasome components.

The second step is the activation phase, in which the different components of the inflammasome are assembled, ultimately allowing for the maturation and release of IL-1β, as a final product. IL-1β significantly enhanced the formation of ASC specks, used as markers of NLRP3 inflammasome activation (Figs. 1C and 1D). This effect was abolished by the NLRP3 inflammasome activation inhibitor MCC 950 (1 µM), thus validating the detection of ASC specks as markers of NLRP3 inflammasome activation (Figs. 1C and 1D). In parallel, an enhanced release of IL-1β to the cell supernatants was observed, that again was mitigated by MCC 950 (Fig. 1E). Altogether, these results prove a positive feedback of the NLRP3 inflammasome activation mediated by IL-1β in human endothelial cells.

**Figure 4.**
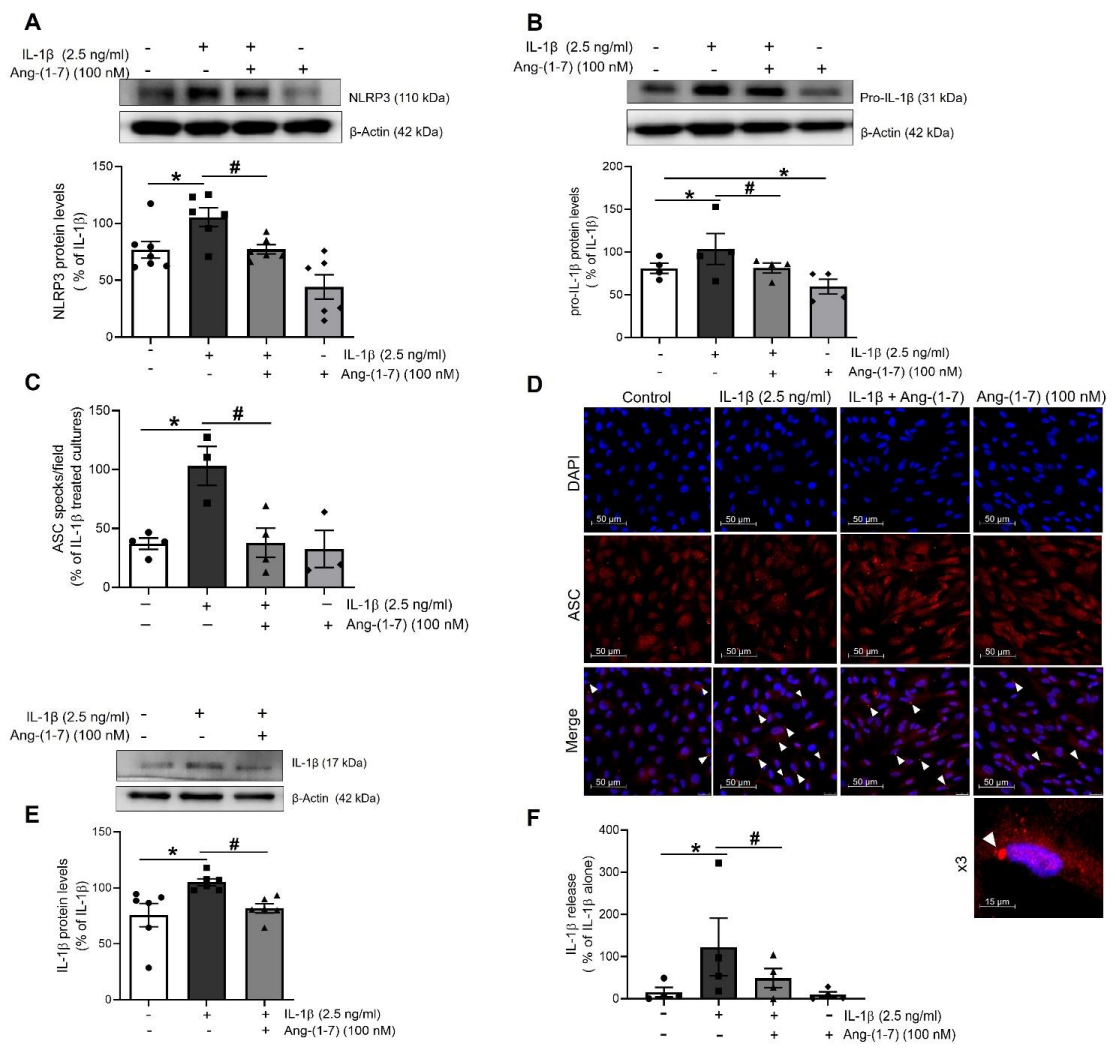
Ang-(1-7) attenuates the induction and activation of the NLRP3 inflammasome mediated by IL-1β in HUVEC. The expression of NLRP3 inflammasome components (A) NLRP3 and (B) pro-IL-1β was determined by Western blot in total lysates from HUVEC stimulated for 18-24 h with IL-1β (2.5 ng/ml) in presence or absence of Ang-(1-7) (100 nM). Representative blots are shown on top of the corresponding graphs, where β-Actin was employed as loading control (n= 6-8; *p<0.05 vs. untreated cells; #p<0.05 vs. IL-1β-treated cells, by two-way ANOVA and Tukey post-hoc test). (C) The NLRP3 inflammasome activation was determined by the detection of ASC specks by indirect immunofluorescence. The percentage of ASC specks per culture field was quantified under a fluorescence microscope (n= 3-4; *p<0.05 vs. untreated cells; #p<0.05 vs. IL-1β-treated cells, by two-way ANOVA and Tukey post-hoc test). (D) Confocal representative photographs (630X magnification) where white arrowheads show the location of toroidal-shaped ASC specks as revealed by a specific antibody against ASC (red), while cell nuclei were counterstained with DAPI (blue). (E) levels of mature IL-1β were measured in total cell lysates by Western blot, and also (F) detected in the supernatants of treated cells by ELISA (n= 3-4; *p<0.05 vs. untreated cells; #p<0.05 vs. IL-1β-treated cells, by two-way ANOVA and Tukey post-hoc test). All data are shown as mean ± SEM and expressed as percentage of IL-1β-induced levels.

**Figure 5.**
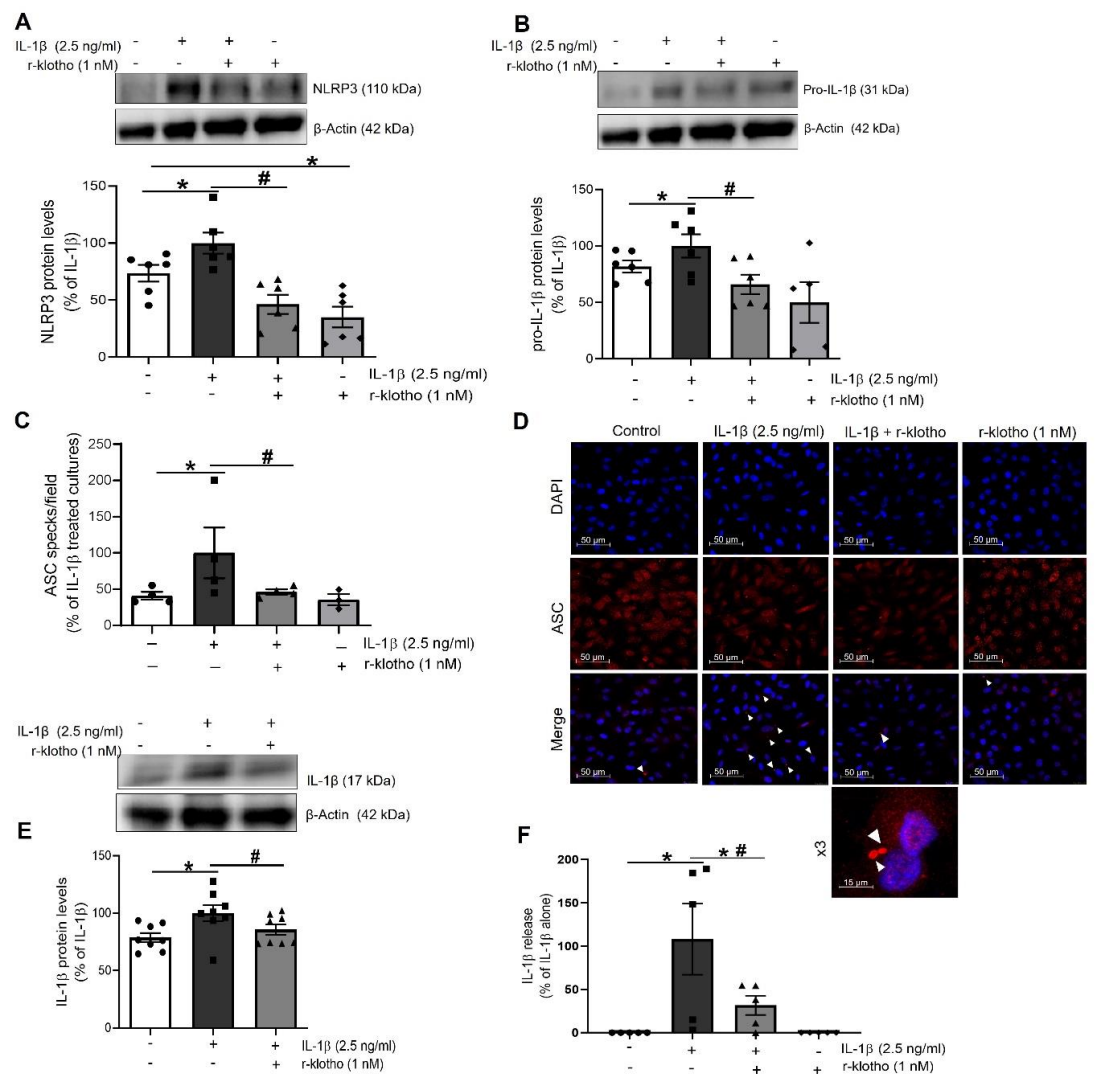
The anti-aging protein klotho mitigates the NLRP3 inflammasome activation mediated by IL-1β in HUVEC. The expression of NLRP3 inflammasome components (A) NLRP3 and (B) pro-IL-1β was determined by Western blot in total lysates from HUVEC stimulated for 18-24 h with IL-1β (2.5 ng/ml) alone or in presence of r-klotho (1 nM). Representative blots are shown on top of the corresponding graphs, where β-Actin was employed as loading control (n= 6-8; *p<0.05 vs. untreated cells; #p<0.05 vs. IL-1β-treated cells, by two-way ANOVA and Tukey post-hoc test). (C) The NLRP3 inflammasome activation was determined by the detection of ASC specks by indirect immunofluorescence. The percentage of ASC specks per culture field was quantified under a fluorescence microscope (n= 3-4; *p<0.05 vs. untreated cells; #p<0.05 vs. IL-1β-treated cultures levels by two-way ANOVA and Tukey post-hoc test). (D) Confocal representative photographs (630X magnification) where white arrowheads mark the location of toroidal-shaped ASC specks and specific antibody against ASC (red) was used, while cell nuclei were counterstained with DAPI (blue). (E) IL-1β levels were measured in total cell lysates by Western blot, and also (F) detected in the supernatants of treated cells by ELISA (n= 4-5; *p<0.05 vs. untreated cells; #p<0.05 vs. IL-1β -treated cells, by two-way ANOVA and Tukey post-hoc test). All data are shown as mean ± SEM and expressed as percentage of IL-1β-induced levels.

### NF-κB mediates the priming and activation of NLRP3 inflammasome by IL-1β

To gain insight into the mechanisms by which IL-1β could favour the priming and activation of NLRP3, we analyzed the role of the transcription factor NF-κB, as a master regulator of inflammatory responses.

NF-κB was activated by IL-1β in HUVEC, as shown by the increased p-p65 levels observed (Fig. 2A), and such activation was blocked by the IL-1R receptors blocker anakinra (1 µg/ml) (Supplementary Fig. 1A).

Moreover, the pharmacological inhibition of NF-κB by pyrrolidine dithiocarbamate (PDTC; 100 µM) totally prevented the increased protein levels of NLRP3, pro-IL-1β and mature IL-1β induced by IL-1β (Figs. 2B, 2C and 2D).

### The positive feedback on NLRP3 inflammasome mediates IL-1β-induced cell senescence in HUVEC

Next, we analyzed whether this positive feedback on the NLRP3 inflammasome could play a role in the endothelial cell senescence induced by IL-1β. In accordance with previous data from our group [10], IL-1β increased the number of SA-β-Gal^+^ in HUVEC cultures (Figs. 3A and 3B), and this effect was dependent on IL-1R receptors since it was abolished by anakinra (Supplementary Fig. 2).

Moreover, IL-1β enhanced the protein levels of the cell cycle inhibitor and senescence marker p21 (Fig. 3C). However, in the presence of the NLRP3 inflammasome inhibitor MCC 950, the stimulation of both SA-β-Gal^+^ cells and p21 by IL-1β was markedly decreased (Figs. 3A, 3B and 3C).

**Figure 6.**
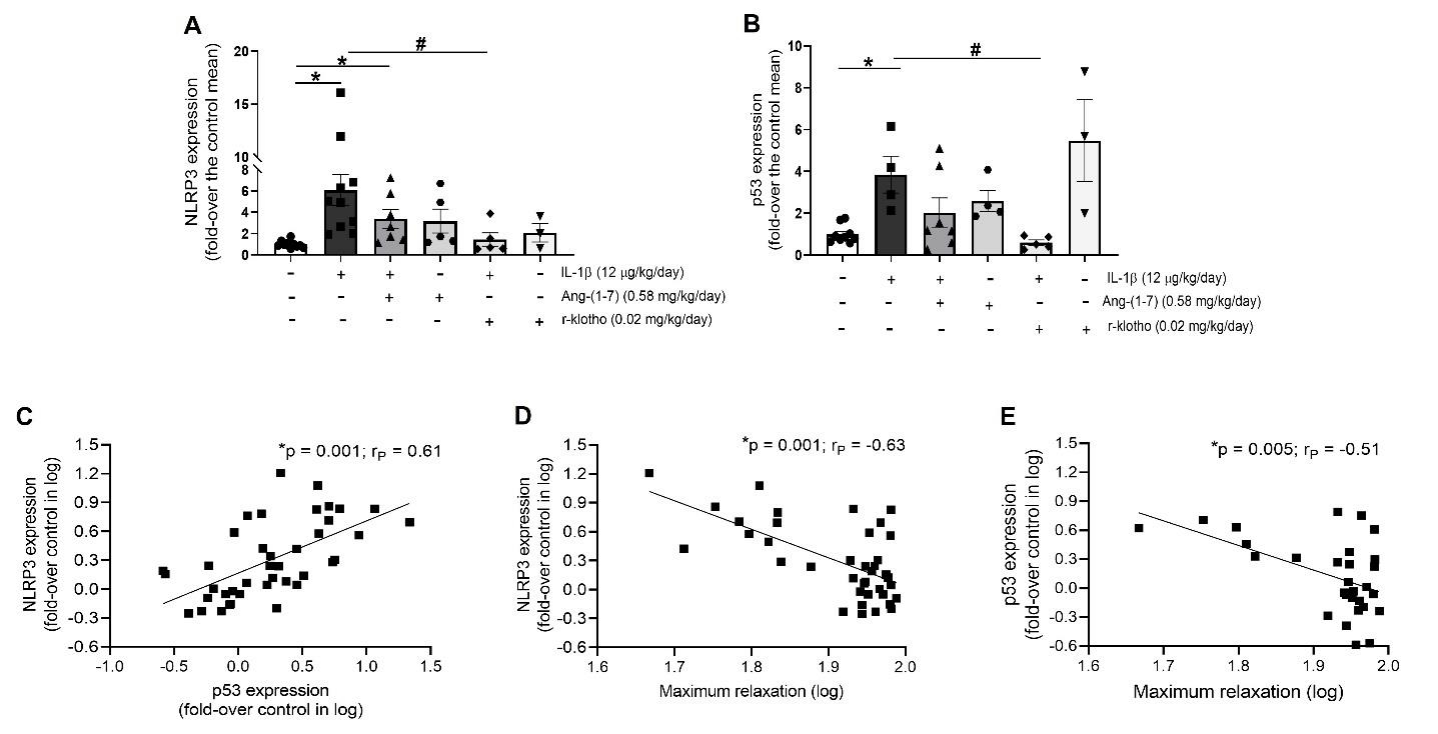
Ang-(1-7) and r-klotho alleviate NLRP3 and p53 expression in aorta from mice treated with IL-1β during 7 days. C57BL6/J mice were treated with IL-1β (12 µg/kg/day) and/or Ang-(1-7) on minipumps (0.58 mg/kg/day) for 7 days. In some cases, IL-1β was combined with i.p. injections of r-klotho (0.02 mg/kg/day) every 2 days. After sacrifice, aortas were isolated and mRNA expression of (A) NLRP3 inflammasome and (B) pro-senescence marker p53 were measured by qRT-PCR. Gene expression was normalized to *18S* rRNA levels. Data are shown as mean ± SEM of 4-9 animals per group and expressed as fold-over the control mean (*p<0.05 vs. untreated mice; #p<0.05 vs. IL-1β by two-way ANOVA and Tukey post-hoc test). Linear correlation analyses of NLRP3 and p53 expression levels with the percentage of maximum relaxation were performed and are expressed in logarithm (*p-value* and Pearson coefficient (r_P_) are indicated on top of each graph).

### Ang-(1-7) mitigates the positive IL-1β-NLRP3 inflammasome feedback in HUVEC

Recently, we have described the protective role of Ang-(1-7) against endothelial senescence promoted by IL-1β [10].

After proving that NLRP3 inflammasome was involved in the endothelial senescence promoted by IL-1β, we sought to explore whether Ang-(1-7) was able to inhibit priming and activation of this multi-protein complex.

In HUVEC, Ang-(1-7) (100 nM) was capable of lowering the expression of NLRP3 (Fig. 4A) and pro-IL-1β (Fig. 4B) proteins, induced by IL-1β.

Furthermore, Ang-(1-7) abolished the NLRP3 inflammasome activation, as shown by a reduction in ASC specks formation (Figs. 4C and 4D) and the subsequent decrease in mature IL-1β protein levels (Fig. 4E) and release (Fig. 4F). Ang-(1-7) was equally capable to prevent NF-κB activation by IL-1β, in line with a major role for this transcription factor in mediating the positive stimulation of NLRP3 inflammasome by IL-1β (Supplementary Fig. 1B).

### r-klotho mitigates the positive feedback on the NLRP3 inflammasome induced by IL-1β in HUVEC

Previously, we reported that Ang-(1-7) exerts its anti-senescence effects in HUVEC via the anti-aging protein klotho [10]. Thus, we assessed whether klotho could itself modulate the priming and activation of the NLRP3 inflammasome in HUVEC.

In IL-1β-treated cells, r-klotho (1 nM) prevented the increased expression of NLRP3 and pro-IL-1β proteins, yielding values even below that of control untreated HUVEC (Figs. 5A and 5B). Similarly, r-klotho inhibited the formation of ASC specks (Figs. 5C and 5D), thus preventing the increased levels of intracellular IL-1β (Fig. 5E) and its release to cell supernatants (Fig. 5F). Similarly, to Ang-(1-7), r-klotho prevented the activation of NF-κB activation by IL-1β (Supplementary Fig. 1C).

### In vivo IL-1β infusion impairs endothelium-dependent responses

After showing that IL-1β promoted endothelial senescence in HUVEC by activation of the NLRP3 inflammasome *in vitro*, we explored the effects of the IL-1β/NLRP3 loop in a more complex *in vivo* environment. Since endothelial senescence is closely linked to endothelial dysfunction, we first explored the impact of the IL-1β-NLRP3 inflammasome loop on endothelium-dependent vascular reactivity.

Mice were infused for 7 days with IL-1β, and after this time period mesenteric artery segments were isolated and tested *ex vivo*. The different treatments had no substantial impact on weight, plasma glucose, or mean arterial pressure values (Supplementary Table 1), nor did they modify the contractile response to NA (Supplementary Table 2).

However, microvascular segments from IL-1β-infused mice exhibited impaired endothelium-dependent vasorelaxation in response to acetylcholine (ACh; 1 nM to 10 μM), which was prevented by the IL-1R blocker anakinra either administered *in vivo* to the mice or added ex vivo to the organ chamber (Supplementary Figs. 3A and Fig. 3B).

**Figure 7.**
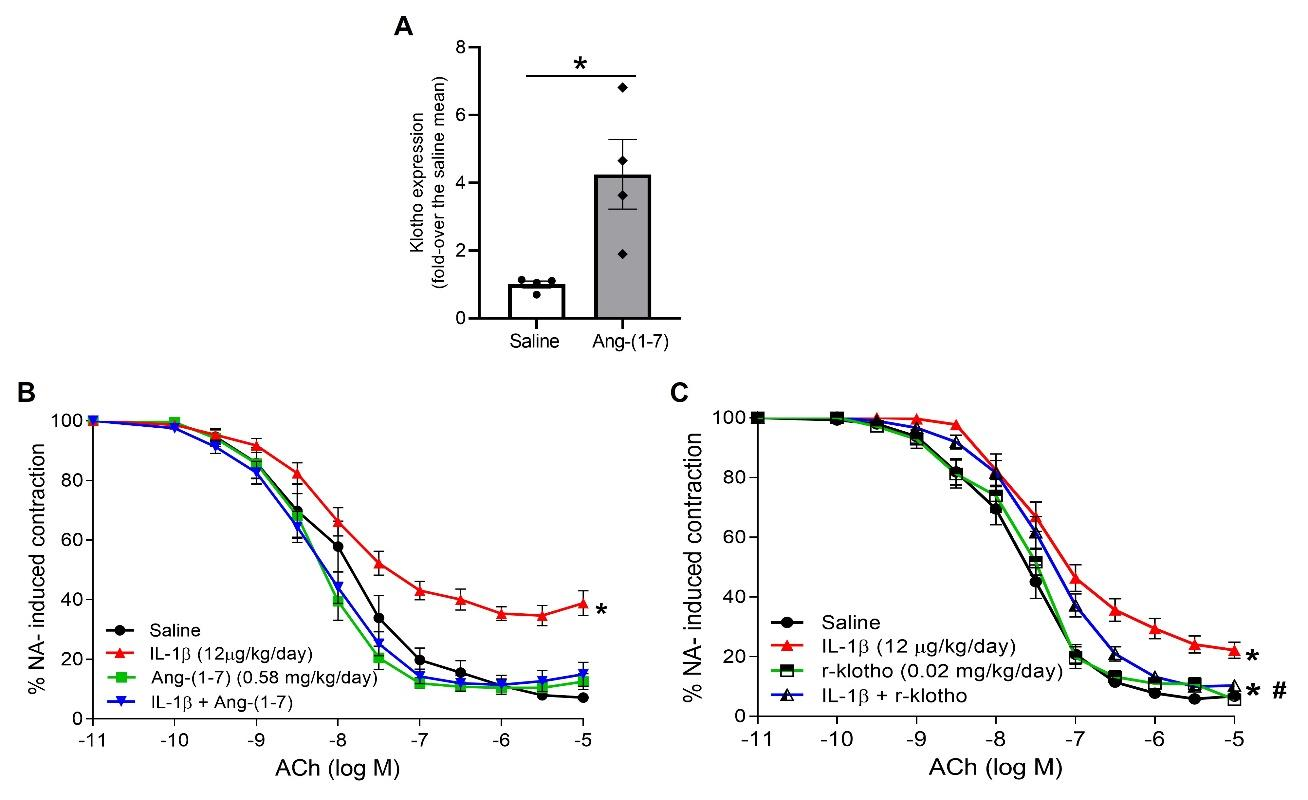
Ang-(1-7) and r-klotho attenuate endothelial dysfunction induced by IL-1β infusion in mice. C57BL6/J mice were treated for 7 days with (A and B) Ang-(1-7) minipumps (0.58 mg/kg/day) and/or (B and C) IL-1β minipumps (12 µg/kg/day). In some cases, IL-1β was combined with i.p. injections of (C) r-klotho (0.02 mg/kg/day) every 2 days. After treatment, mesenteric microvessels were isolated and vascular relaxation was tested. The microvessels were pre-contracted with 3 µM noradrenaline (NA) and submitted to cumulative concentrations of the endothelium-dependent vasodilator acetylcholine (ACh, 100 pM - 10 µM). Values (mean ± SEM) in contraction curves were calculated as average percentage of the previous NA-evoked contraction for all segments coming from 4-9 mice per group (*p<0.05 vs. control untreated segments response, #p<0.05 vs. IL-1β-induced response by two-way ANOVA followed by Fisher’s LSD test). (A) In the case of mice treated with Ang-(1-7) minipumps, aortas were isolated and *Klotho* mRNA levels were measured by qRT-PCR and normalized to *18S* rRNA levels. Data are shown as mean ± SEM of 4 animals per group and expressed as fold-over the control mean (*p<0.05 vs. untreated mice by unpaired t-test).

**Figure 8.**
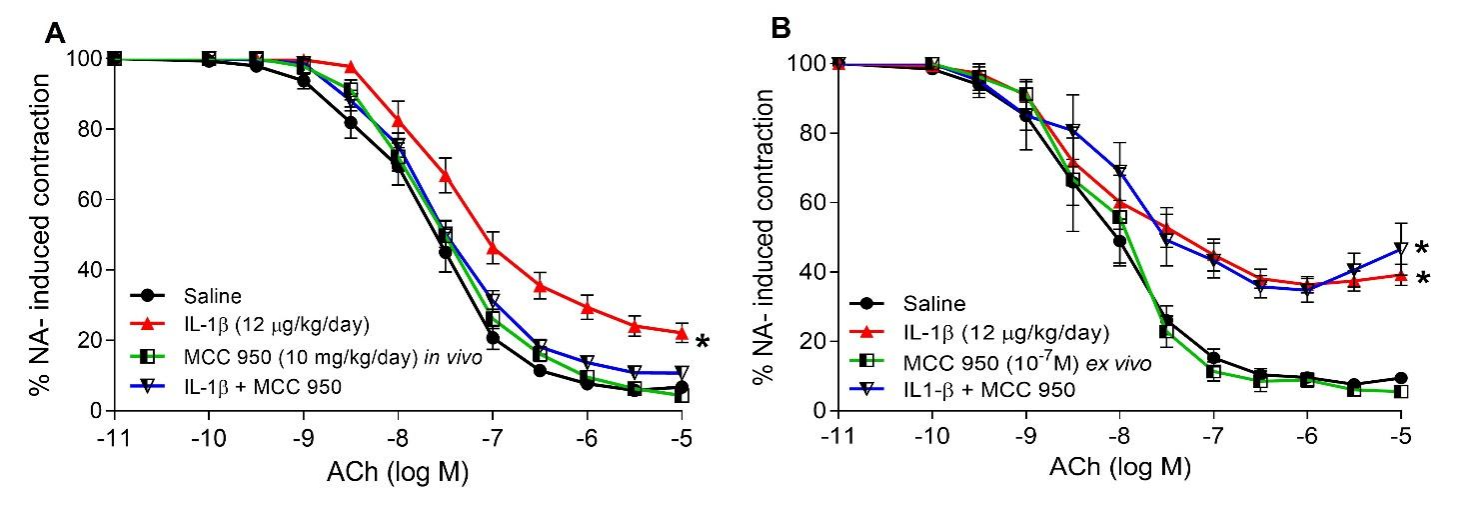
NLRP3 inflammasome blockade alleviates endothelial dysfunction induced by IL-1β infusion in mice. C57BL6/J mice were treated for 7 days with IL-1β minipumps (12 µg/kg/day) and/or combined with i.p. injections of (A) MCC 950 (10 mg/kg/day) every 2 days. After treatment, mesenteric microvessels were isolated and vascular relaxation was tested. The microvessels were pre-contracted with 3 µM NA and submitted to cumulative concentrations of the endothelium-dependent vasodilator ACh (100 pM to 10 µM) in the microvessels pre-incubated with (B) MCC 950 (10^-7^ M). Values (mean ± SEM) in contraction curves were calculated as average percentage of the previous NA-evoked contraction for all segments coming from 4-9 mice per group (*p<0.05 vs. control untreated segments response by two-way ANOVA followed by Fisher’s LSD test).

### IL-1β infusion augments the vascular expression of NLRP3 and p53

Since we had shown that IL-1β promoted the *in vitro* expression of NLRP3 components in human cultured endothelial cells, we analyzed whether such effect could also be observed *in vivo* in mice. The infusion of IL-1β for 7 days promoted an over-expression of NLRP3 mRNA in aortic tissue as compared with control levels (Fig. 6A). Moreover, this over-expression of NLRP3 was accompanied by enhanced levels of senescence markers, as evidenced by increased p53 expression (Fig. 6B), with a positive correlation observed between these two parameters (Fig. 6C). Interestingly, a negative association of both NLRP3 and p53 mRNA levels with microvascular reactivity, in terms of the maximal relaxation achieved in response to ACh, was observed (Figs. 6D and 6E).

### Ang-(1-7) and r-klotho attenuate NLRP3 and p53 over-expression and endothelial dysfunction induced by IL-1β infusion in vivo

The *in vivo* administration of Ang-(1-7) was capable to mitigate the enhanced expression of aortic NLRP3 and p53 induced by IL-1β (Figs. 6A and 6B). Interestingly, Ang-(1-7) also augmented the vascular expression of the anti-aging protein klotho (Fig. 7A). In fact, r-klotho administration mimicked the preventive effect of Ang-(1-7) on the over-expression of NLRP3 and p53 induced by IL-1β (Figs. 6A and 6B). When infused over the *in vivo* treatment period with IL-1β, both Ang-(1-7) and r-klotho prevented the endothelial dysfunction induced by the cytokine (Figs. 7B and 7C).

### NLRP3 blockade prevents the endothelial dysfunction induced by IL-1β infusion

Finally, we addressed the impact of *in vivo* over-expression of NLRP3 inflammasome on the impaired vascular reactivity induced by IL-1β. The blockade of NLRP3 assembly with MCC 950 during the 7 days of infusion with the cytokine completely preserved the vasorelaxant responses (Fig. 8A). However, MCC 950 had no effect when administered *ex vivo* in isolated vascular segments from IL-1β treated animals (Fig. 8B), indicating that this drug was not effective against an already activated NLRP3 inflammasome.

## DISCUSSION

IL-1β is one of the main mediators of the inflammatory response involved in several vascular pathologies [16-19]. Blockage of this cytokine by the monoclonal antibody canakinumab has recently shown to exert protective effects against CV complications, as revealed by the CANTOS clinical trial [20, 21]. These results have been crucial to emphasize the importance of inflammation in the pathogenesis of CV diseases. Recently, our group described the pro-senescent effects of IL-1β on endothelial human cells [10], pointing out at a novel mechanism by which chronic inflammation impacts on aging. These results have put IL-1β in the spotlight of sterile log-term inflammation associated with aging, the so-called inflammaging process [22]. All these findings suggest a potential benefit of IL-1β-inhibitory therapies in the management of vascular aging, although further understanding of the mechanisms linking inflammation, IL-1β, endothelial dysfunction and cell senescence is needed. In this paper, we have deepened into the mechanisms by which IL-1β promotes endothelial senescence and linked them to *in vivo* vascular dysfunction. Our *in vitro* results show a positive feedback loop by which IL-1β, the final product of the NLRP3 inflammasome, activates this multicomplex protein and increases the levels of its major components, inducing its assembly which finally results in the promotion of the cytokine's own release. This positive stimulation of IL-1β on the NLRP3 inflammasome appears to be driven by the intracellular activation of NF-κB, a master player in inflammatory responses, in line with previous results from other groups in other cell types [23, 24]. The activation of NF-kB is induced via IL-1R as evidenced after pharmacologically blocking the receptor with anakinra.

Importantly, the pharmacological inhibitor of the NLRP3 inflammasome assembly MCC 950 mitigated the pro-senescent effect of IL-1β, proving the involvement of the hereby described loop in human endothelial cell senescence. To our knowledge, this is the first description that this positive feedback loop is causing cell senescence in any cell or tissue. Although NLRP3 inflammasome is a key component of the innate immune system, recent evidence suggests it is also critical for metabolic homeostasis [22, 25, 26, 27]. Indeed, over-activation of this multi-complex protein has been related to sterile chronic inflammation associated with aging and metabolic syndromes [22] while *Nlrp3* gene deficiency promotes healthspan in aging mice [28].

Endothelial cell senescence has been reported to promote NLRP3 inflammasome activation and subsequent IL-1β release both *in vitro* and *in vivo* [29]. Our results suggest that NLRP3 inflammasome activation is a cause but also a consequence of endothelial cell senescence. Aged individuals exhibit increased levels of IL-1β as a feature of the chronic, low-grade inflammation associated with aging [22, 30]. According to our data, this would trigger the activation of the NLRP3 inflammasome and further release of IL-1β, with this loop boosting cell senescence and SASP, leading to a vicious circle underlying endothelial senescence propagation and vascular aging progression.

From a functional point of view, our *in vivo* results also prove a direct implication of IL-1β in the development of defective vasorelaxation by means of the NLRP3 inflammasome. Furthermore, gene expression analyses showed the vascular induction of mRNAs for NLRP3 and the senescence marker p53 by IL-1β, as well as a negative correlation of these parameters with the vasorelaxation capability. Consistently, it has been described that the accumulation of senescent cells promotes an inflammatory environment that might lead to endothelial dysfunction, together with a decrease in nitric oxide bioavailability [31, 32].

Globally, these results point at NLRP3 inflammasome as a main mediator of endothelial dysfunction and premature vascular aging in those pathological conditions in which excess IL-1β may impact the vasculature. This can be the case for metabolic diseases, such as metabolic syndrome, obesity, and type 2 diabetes mellitus (T2DM), as well as some auto-inflammatory disorders [33-35], which may benefit from IL-1β blockers, or even NLRP3 inflammasome inhibitory therapies.

In search for antisenescence pharmacological approaches, we have recently described an anti-senescence effect of the heptapeptide Ang-(1-7) in human endothelial cells, mediated by Mas receptors and klotho induction [10, 36]. Ang-(1-7) has also been shown to induce miRNAs that could inactivate the NLRP3 inflammasome [37, 38] and prevent liver fibrosis mediated by the NLRP3 inflammasome [39]. Concerning klotho, it has been described to attenuate the NLRP3 inflammasome induction in the choroid plexus of diabetic mice [38]. In this study, both Ang-(1-7) and klotho inhibited NF-κB activation by IL-1β and proved to effectively protect against vascular NLRP3 inflammasome over-activation and the subsequent onset of endothelial cell senescence and vascular dysfunction.

Thus, we here provide further insight on the mechanisms by which Ang-(1-7) and klotho may act as anti-aging agents. Beyond aging-associated vascular dysfunction, these two molecules may also have clinical utility to treat other pathologies triggered by NLRP3 inflammasome over-activation [15]. By preventing inflammaging and endothelial senescence, Ang-(1-7) and klotho also represent novel alternative therapies to treat CV aging-related diseases. Supporting this hypothesis, we and others have shown robust anti-inflammatory effects for Ang-(1-7) in the vascular smooth-muscle cell type [29, 40], which reinforce the therapeutic potential of this protective branch of the RAS for preventing or treating vascular disorders. Indeed, Ang-(1-7)-based drugs that are currently being assayed in clinical trials could be approved for clinical use in the near future [22, 41, 42].

In conclusion, this study reveals the auto-activation of NLRP3 inflammasome as a key mechanism by which IL-1β promotes endothelial cell senescence and aging-associated vascular damage. In addition, the RAS heptapeptide Ang-(1-7) and the anti-aging protein klotho arise as novel potential therapies to inhibit the deleterious effects of the NLRP3 inflammasome/IL-1β loop in vascular pathological settings.

## Supplementary Materials

The Supplementary data can be found online at: www.aginganddisease.org/EN/10.14336/AD.2021.0617.


