# Supplementary material for: Pharmacological Blockade of NLRP3 Inflammasome/IL-1β-Positive Loop Mitigates Endothelial Cell Senescence and Dysfunction
Source: Aging Dis. 2022 Feb 1;13(1):284–97. doi: 10.14336/AD.2021.0617 (PMC8782550; doi:10.14336/AD.2021.0617)
Supplement: Supplementary file 1 [file AD-13-1-284-s.pdf]

SUPPLEMENTARY DATA

Pharmacological Blockade of NLRP3 Inflammasome/IL-1β-Positive Loop Mitigates Endothelial Cell Senescence and Dysfunction

Alejandra Romero[1,2,#], Pilar Dongil[1,2,#], Inés Valencia[1,2,3], Susana Vallejo[1,2], Álvaro San Hipólito-Luengo[1,2], Guillermo Díaz-Araya[4,5], José L. Bartha[2,6], María M. González-Arlanzón[6], Fernando Rivilla[7], Fernando de la Cuesta[1,2,*], Carlos F. Sánchez-Ferrer[1,2, †,*], Concepción Peiró[1,2, †,*]

© 2021. Romero A & Dongil P et al. Published online at http://www.aginganddisease.org/EN/10.14336/AD.2021.0617

SUPPLEMENTARY DATA

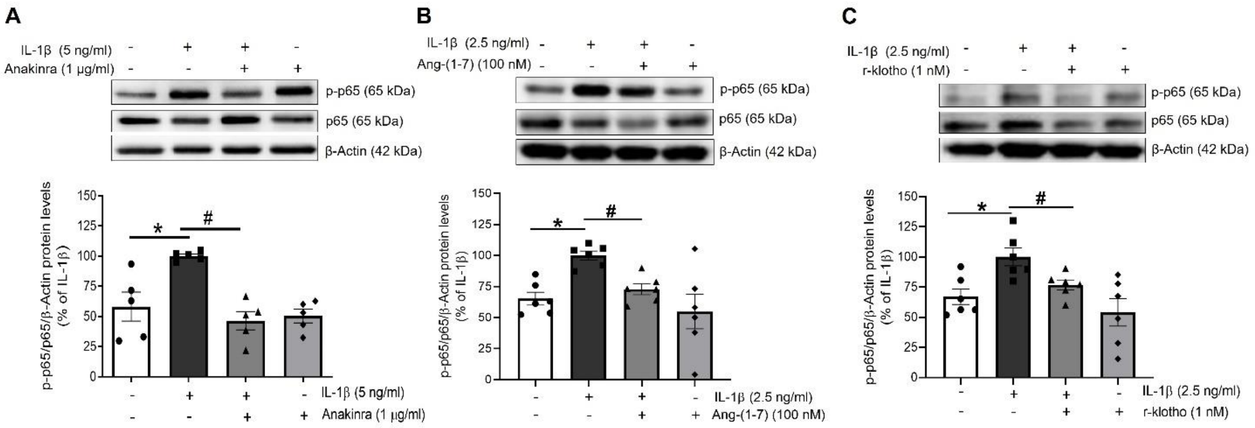

Supplementary Figure 1. NF-kB activation is abrogated by both anakinra, Ang-(1-7) and r-klotho in endothelial human cells. HUVEC were treated with IL-1β (2.5 ng/ml) alone or in presence of **(A)** Anakinra (1 μg/ml), **(B)** Ang-(1-7) (100 nM) or **(C)** r-klotho (1 nM) for 18 h. The expression of p-p65 and total p65 were determined by Western blot in total lysates from HUVEC. A representative blot is shown on top of each graph, where β-Actin was employed as loading control (n= 5-6; *p<0.05 vs. untreated cells; #p<0.05 vs. IL-1β-treated cells, by two-way ANOVA and Tukey post-hoc test). All data are shown as mean ± SEM and expressed as percentage of IL-1β-induced levels.

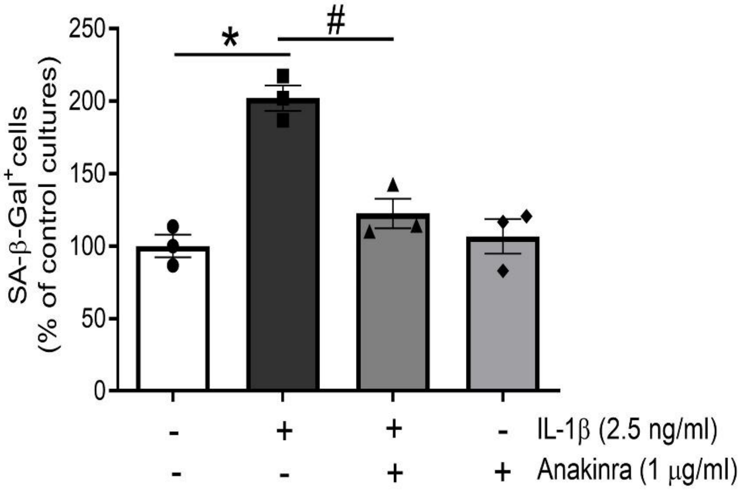

Supplementary Figure 2. **Anakinra blocks the pro-senescence effect of IL-1β in endothelial human cells.** HUVEC were treated with IL-1β (2.5 ng/ml) alone or in presence of Anakinra (1 μg/ml) for 24 h. SA-β-Gal positive stained cells were quantified by manual scoring by a blind observer after the indicated treatments. Data are expressed as percentage of the number of senescent cells stained in non-treated cells (n= 3; *p<0.05 vs. untreated cells; #p<0.05 vs IL-1β-treated cultures levels by two-way ANOVA and Tukey post-hoc test). All data are shown as mean ± SEM and expressed as percentage of control cultures levels.

© 2021. Romero A & Dongil P et al. Published online at http://www.aginganddisease.org/EN/10.14336/AD.2021.0617

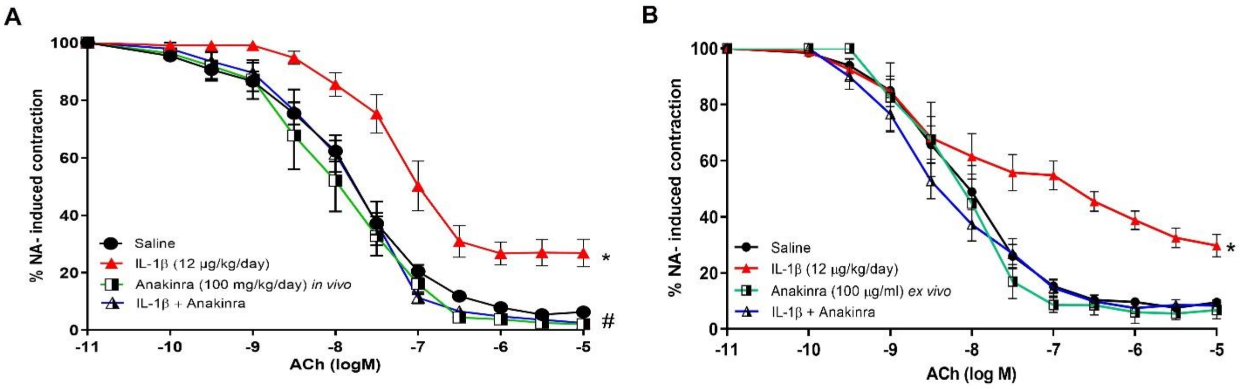

Supplementary Figure 3. The chronic treatment with IL-1β deteriorates the microvascular relaxation in mice. C57BL6/J mice were treated with minipumps of IL-1β (12 µg/kg/day) for 7 days and/or with **(A)** anakinra (100 mg/kg/day), the mesenteric microvessels were isolated and the vascular relaxation was tested. The microvessels were pre-contracted with 3 µM NA and submitted to cumulative concentrations of the endothelium-dependent vasodilator ACh (100 pM to 10 µM) in the microvessels pre-incubated with **(B)** anakinra (100 µg/ml). Values (mean ± SEM) in contraction curves were calculated as average percentage of the previous NA-evoked contraction for all segments coming from 4-5 mice per group. *$p<0.05$ vs. control untreated; #$p<0.05$ vs. IL-1β-induced response by two-way ANOVA followed by Fisher's LSD test

Supplementary methods Table 1. Sequences of primers used in the different gene expression assays by RT-qPCR.

**Gene Name**	***Forward primer (5'-3')***	***Reverse primer (5'-3')***
Klotho	CAAAGTCTTCGGCCTTGTTC	CTCCCCAAGCAAAGTCACA
Nlrp3	AGAAGAGACCACGGCAGAAG	CCTTGGACCAGGTTCAGTGT
p53	GTATTTCACCCTCAAGATCC	TGGGCATCCTTTAACTCTA
18s rRNA	GGAAGGGCACCACCAGGAGT	TGCAGCCCCGGACATCTAAG

© 2021. Romero A & Dongil P et al. Published online at http://www.aginganddisease.org/EN/10.14336/AD.2021.0617

SUPPLEMENTARY DATA

Supplementary Table 1. Average weight, basal glucose and arterial pressure before and after treatment administration to mice.

	**Average weight (g)**		**Plasma glucose (mg/dL)**		**Arterial pressure (mmHg)**	
***ex vivo* administered drugs**	**Day 0**	**Day 7**	**Day 0**	**Day 7**	**Day 0**	**Day 7**
Saline	28.4 ± 0.6	27.6 ± 0.4	99.5 ± 3.7	104.5 ± 3.8	100.6 ± 3.5	98.5 ± 3.1
IL-1β	27.7 ± 0.3	25.3 ± 0.5*	116.5 ± 4.7	88.6 ± 2.9*	103.8 ± 4.1	103.9 ± 3.3
IL-1β + Ang-(1-7)	26.3 ± 0.4	25.8 ± 0.4	105.1 ± 5.1	87.5 ± 4.2*	100.2 ± 8.3	98.7 ± 3.8
IL-1β + r-klotho	27.8 ± 0.9	26.5 ± 1.1	97.2 ± 0.8	109.4 ± 4.0*	99.6 ± 2.5	101.4 ± 1.8
IL-1β + MCC 950	26.3 ± 0.6	24.9 ± 0.6	101.6 ± 3.4	88.4 ± 2.8	101.9 ± 2.8	98.7 ± 2.8
Ang-(1-7)	26.4 ± 1.5	27 ± 1.6	101.6 ± 5.8	109.6 ± 5.7	105.1 ± 8.3	104.5 ± 6.5
r-klotho	25.4 ± 1.8	25.2 ± 1.7	98.6 ± 7.1	98.3 ± 6.6	103.2 ± 2.9	102.4 ± 3.4
MCC 950	27.2 ± 0.9	25.7 ± 1.1	108.3± 1.7	104.3 ± 1.9	99.7 ± 2.5	102.7 ± 2.2

Data are represented as mean ± SEM, 4-9 mice for each group. *p<0.05 vs. Day 0, according to two-way ANOVA followed by Fisher's LSD test.

Supplementary Table 2. Values of strength by NA-induced contraction prior to relaxation, mean pEC_{50} value and maximum relaxation.

***ex vivo* administered drugs**	**Tone prior to relaxation**	**Mean pEC_{50} value**	**Maximum relaxation (%)**
Saline	5.1 ± 0.6	8.0 ± 0.5	90.5 ± 1.6
IL-1β	5.9 ± 0.4	7.6 ± 1.0	66.6 ± 3.2*
IL-1β + MCC 950	4.9 ± 0.5	7.7 ± 0.9	53.4 ± 7.5*
IL-1β + Anakinra	4.7 ± 0.6	8.4 ± 0.5	92.3 ± 2.0#
MCC 950	5.7 ± 2.3	8.0 ± 0.5	94.4 ± 1.5
Anakinra	5.3 ± 2.2	8.2 ± 0.5	93.2 ± 3.1#

Data are represented as mean ± SEM, 4-9 mice for each group. *p<0.05 vs. saline, #p<0.05 vs. IL1-β-induced response by two-way ANOVA followed by Fisher's LSD test.

© 2021. Romero A & Dongil P et al. Published online at http://www.aginganddisease.org/EN/10.14336/AD.2021.0617
