# Supplementary figures and images for: Pharmacological Blockade of NLRP3 Inflammasome/IL-1β-Positive Loop Mitigates Endothelial Cell Senescence and Dysfunction
Source: Aging Dis. 2022 Feb 1;13(1):284–97. doi: 10.14336/AD.2021.0617 (PMC8782550; doi:10.14336/AD.2021.0617)

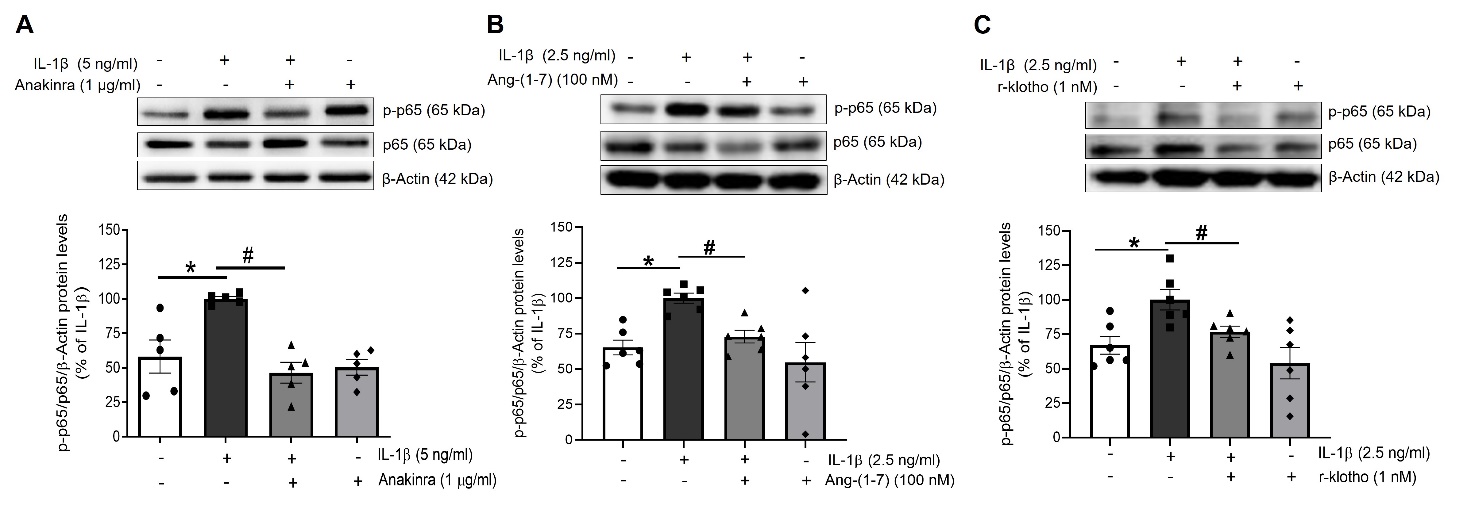

Supplement: Supplementary file 2 [file ad-13-1-284-s-g1.jpg]

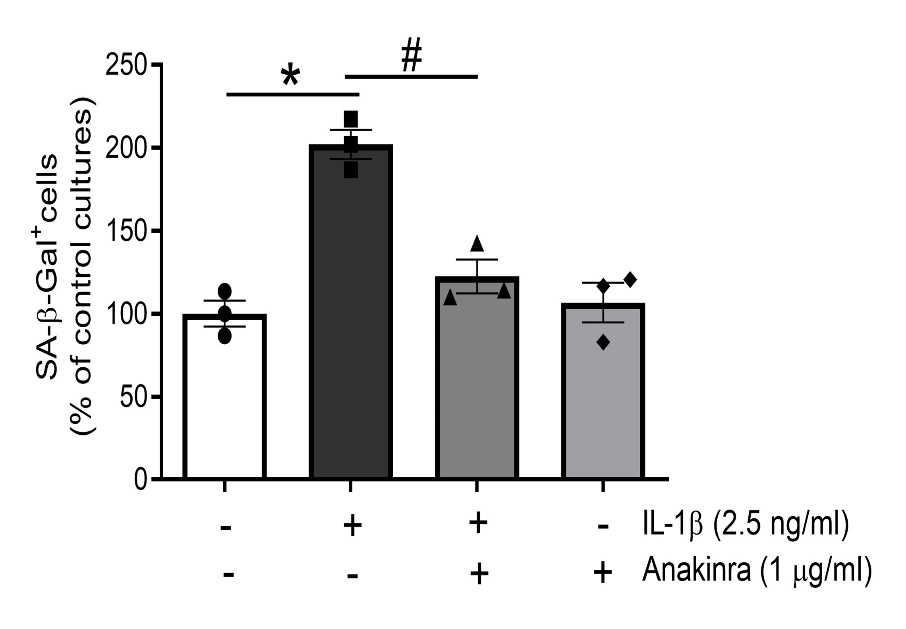

Supplement: Supplementary file 3 [file ad-13-1-284-s-g2.jpg]

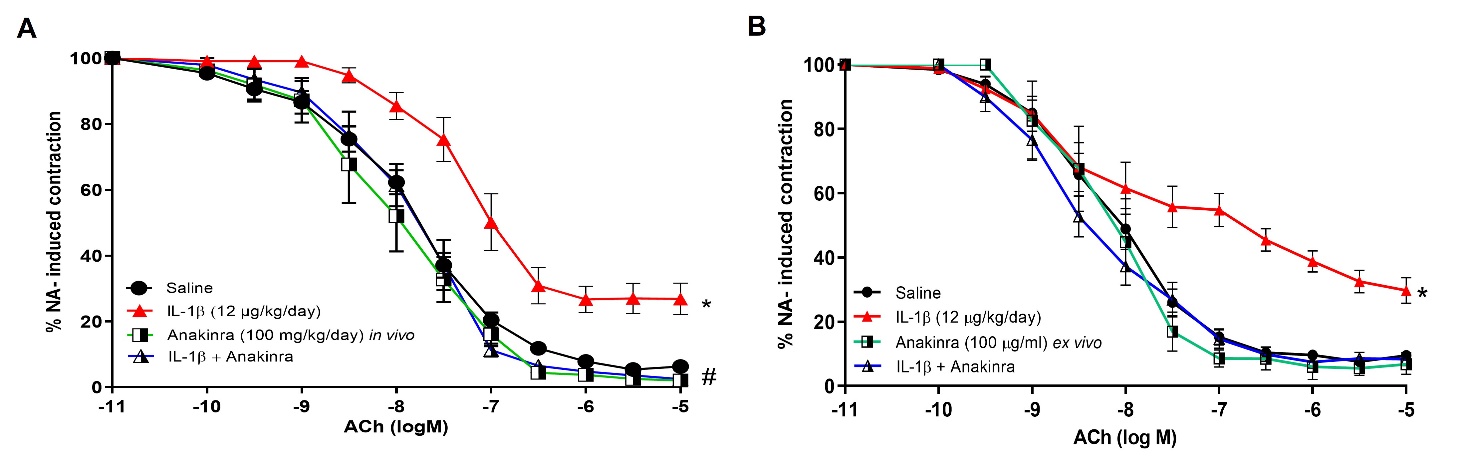

Supplement: Supplementary file 4 [file ad-13-1-284-s-g3.jpg]
